# How many challenges we may encounter in anterior megalophthalmos with white cataract: a case report

**DOI:** 10.1186/s12886-019-1133-y

**Published:** 2019-05-30

**Authors:** Ao Miao, Keke Zhang, Jifeng Yu, Wenwen He, Yi Lu, Xiangjia Zhu

**Affiliations:** 1 Eye Institute, Eye and ENT Hospital, Shanghai Medical College, Fudan University, Shanghai, 200031 China; 2 Department of Ophthalmology and Visual Science, Eye and ENT Hospital, Shanghai Medical College, Fudan University, Shanghai, China; 3 NHC Key Laboratory of Myopia (Fudan University), Shanghai, China; 4 Key Laboratory of Myopia, Chinese Academy of Medical Sciences, Shanghai, China; 5 Department of Ophthalmology, Beijing Children’s Hospital, Capital Medical University, National Center for Children’s Health, Beijing, China

**Keywords:** Anterior megalophthalmos, Cataract, Intraocular lens, Dislocation, IOL power calculation, Haigis formula

## Abstract

**Background:**

Anterior megalophthalmos is a rare congenital disease which mainly features enlargement of the anterior segment. Cataract surgeries in anterior megalophthalmos can be challenging due to the anatomical anomalies while the studies upon the surgical design have been less integrated.

**Case presentation:**

A 37-year-old woman presented with progressively blurred vision in the right eye after a transient fever 10 months ago. Her ocular history included hypermetropia with a spherical equivalent of + 4.00 OU. The review of systems showed bilateral varus deformity of distal interphalangeal joints on the little fingers. The patient denied family history of hereditary ocular diseases and her sister was born with uterus didelphys. On initial examinations, the corrected distance visual acuity was hand motion OD and 20/33 OS. Her intraocular pressure was 15 mmHg OD and 16 mmHg OS. Horizontal corneal diameter was 14 mm OD and 13.88 mm OS and axial length was 24.87 mm OD and 25 mm OS. Anterior segment photography showed bilateral iridal atrophy with deficiency in pupillary dilation and white cortically mature cataract in the right eye. Inspection by anterior segment optical coherence tomography indicated bilateral augmented anterior chambers with backward iridal concave on horizontal orientation. Ultrasound biomicroscopy showed partially peripheral anterior synechiae and pectinate ligaments at chamber angle in both eyes and opacified lens with the apparently elongated suspensory ligaments in the right eye. A deliberately selected 1-piece foldable intraocular lens (IOL) with frame haptics was implanted after phacoemulsification for good IOL stability. During the follow-up, the visual rehabilitation appeared relatively good and a lower degree of IOL dislocation comparing with existing reports was verified by OPD-Scan III aberrometry.

**Conclusions:**

We presented the challenges and the original findings from a case of congenital anterior megalophthalmos with white cataract who underwent phacoemulsification and IOL implantation. This is the first report describing the comparison of the different IOL power calculation formulas in anterior megalophthalmos. Compared to the SRK/T and the Holladay II formulas, the Haigis formula could be a more accurate choice for the IOL calculation in anterior megalophthalmos according to our case. Moreover, the deliberate selection of IOLs is essential for IOL stability in these patients.

## Background

Anterior megalophthalmos is a rare congenital disease which mainly features bilateral megalocornea, broad deep anterior chambers and ciliary ring enlargement with quite normal axial length (AL) [1–3]. The pathogenesis relates mostly to hereditary factors while the mechanism remains unclear. Since the congenital lesions of anterior megalophthalmos are always nonprogressively asymptotic and easy to be overlooked, onset of cataract and lens subluxation are the most common initial symptoms of this disease. Other complications may include anterior embryotoxon, mosaic corneal dystrophy, iris atrophy, spontaneous vitreous hemorrhage, retinal detachment, and peripheral retinal degenerations [4–6].

Cataract surgeries on patients with anterior megalophthalmos are challenging. Deficiency of pupillary dilation can always be observed due to iris atrophy [1]. Zonular anomalies and enlarged sulcus will also highly increase the risk of postoperative intraocular lens (IOL) dislocation at the same time [7]. For the anatomical anomalies of anterior megalophthalmos, deliberate selection of IOLs and power calculation formulas before the cataract surgery are vital for small postoperative refractive error and matter a lot to visual rehabilitation. In addition, a more careful follow-up is necessary for patients with anterior megalophthalmos to prevent postoperative complications [8].

We report a challenging case of anterior megalophthalmos accompanied with secondary white cataract (cortically mature) who underwent phacoemulsification and in-the-bag IOL implantation successfully, highlighting the rather good visual rehabilitation by deliberate selection of IOLs and postoperative follow-up.

## Case presentation

A 37-year-old woman presented to our hospital with complaints of blurred vision in the right eye for 10 months. She had no family history of hereditary ocular diseases and no previous history of eye surgery or ocular trauma. In addition, the patient had bilateral varus deformity of distal interphalangeal joints on the little fingers. Also, her sister was born with uterus didelphys.

### Preoperative examinations

#### Routine inspections

The best corrected visual acuity (BCVA) was hand motion in the right eye and 20/33 in the left eye. The refraction was + 4.25/− 0.50 × 90 in the left eye. Horizontal corneal diameters were apparently enlarged in both eyes (the right eye 14 mm/ the left eye 13.88 mm). Applanation intraocular pressure (IOP) were normal in both eyes. Main measures of the present case are summarized in Table 1.

**Table 1:** Main preoperative measures of the present case

Quantitative measurements	OD	OS
AL	24.87 mm	25.00 mm
LT	5.23 mm	3.60 mm
ACD	2.644 mm	3.677 mm
WTW	14.00 mm	13.88 mm
Chamber Angle	36.0°	76.8°
CCT	535 um	537 um
K1/K2	39.2 D /40.3 D	39.1 D/39.8 D
Applanation IOP	15 mmHg	16 mmHg
Count of corneal endothelium	2544/mm^2^	2821/mm^2^

*AL* axial length, *LT* lens thickness, *ACD* anterior chamber depth, *WTW* white to white, *CCT* central corneal thickness, *IOP* intraocular pressure

#### Special examinations

**Fig. 1:**
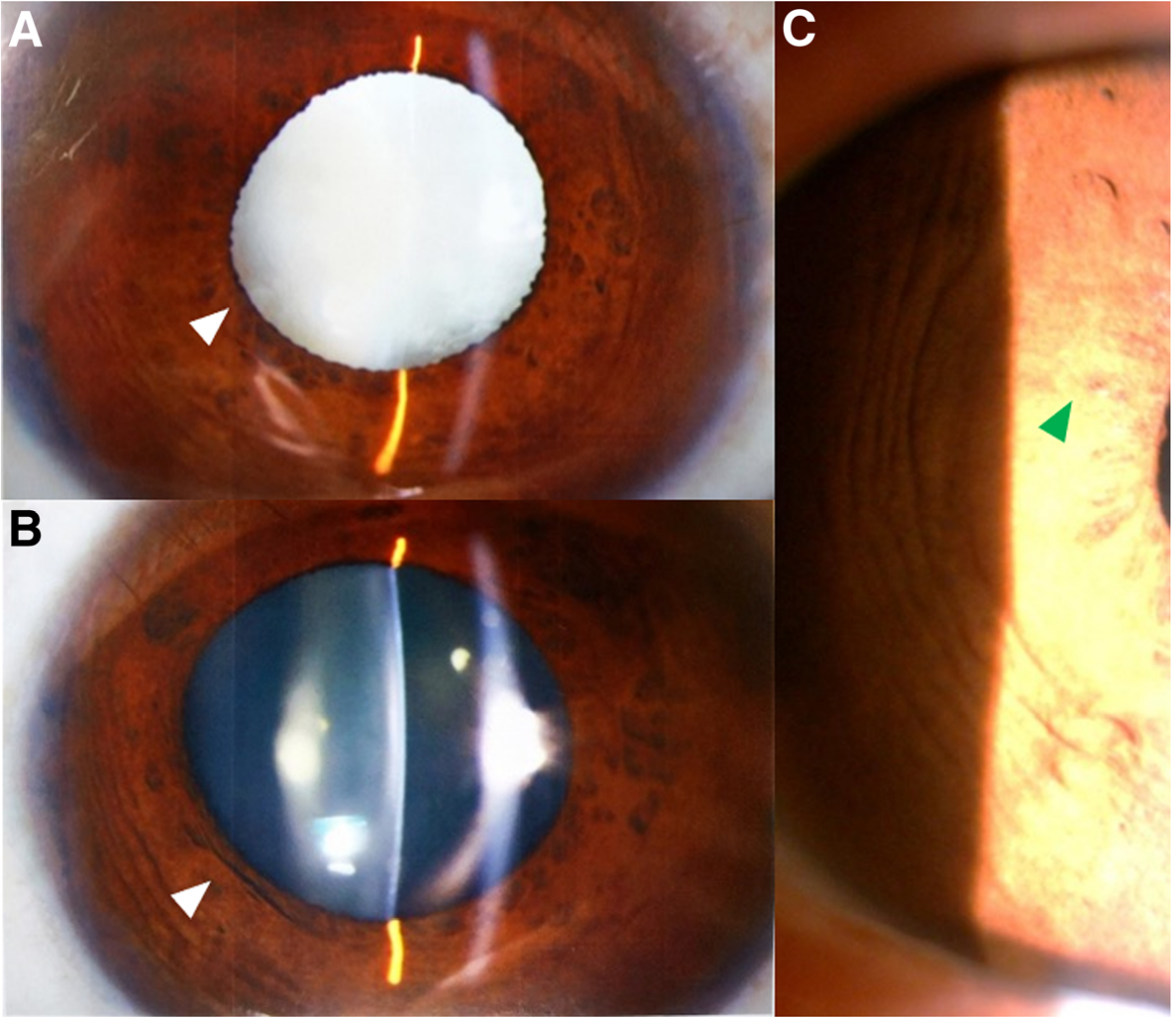
Preoperative slit-lamp examination. **a**, **b** Preoperative slit-lamp examination of the right eye and the left eye showing enlarged corneas, deep anterior chambers, insufficiently dilated pupils (indicated with white arrowheads) and white cataract in the right eye. **c** Preoperative slit-lamp photography of the right eye showing mild concomitant iridal atrophy (indicated with green arrowhead)

**Fig. 2:**
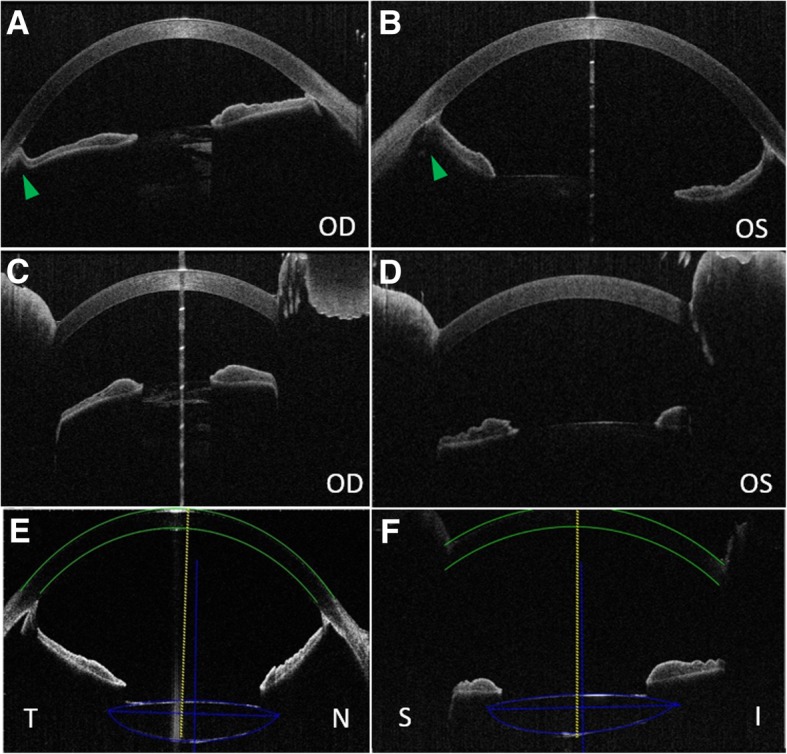
Preoperative and postoperative anterior segment optical coherence tomography (AS-OCT). **a**, **b** Preoperative AS-OCT on horizontal orientation verifying augmented anterior chambers in both eyes with backward iridal concavity in the left eye and markedly reduced backward iridal concavity resulted from the swelling cataractous lens in the right eye (indicated with green arrowheads). **c**, **d** Preoperative AS-OCT on vertical orientation of the right eye and the left eye verifying augmented anterior chambers with rather flat iris. **e** Postoperative AS-OCT inspection of the right eye on horizontal orientation showing slight decentration of IOL and backward iridal concavity. **f** Postoperative AS-OCT inspection of the right eye on vertical orientation showing slight decentration of IOL and flat iris

**Fig. 3:**
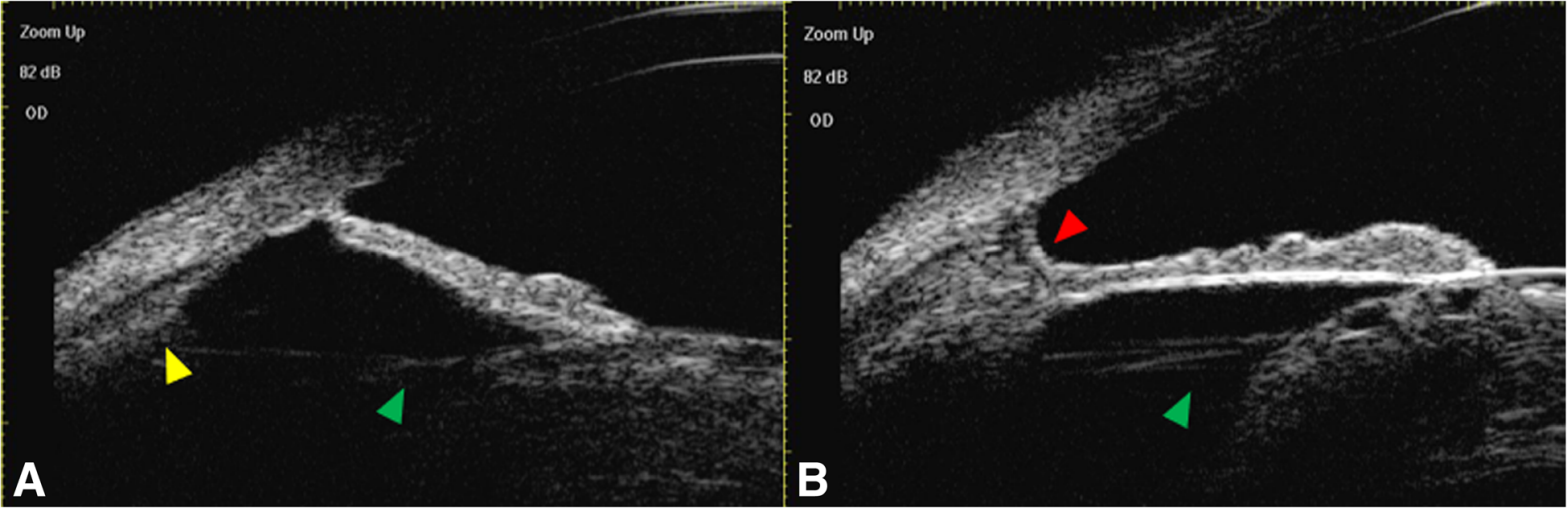
**a**, **b** Preoperative ultrasound biomicroscopy of the right eye. Preoperative ultrasound biomicroscopy of the right eye showing the cloudy cataractous lens suspended to the small ciliary processes (indicated with yellow arrowhead) by the apparently elongated suspensory ligaments (indicated with green arrowheads) caused by congenital enlargement of the anterior segment. The pectinate ligament (indicated with red arrowhead) was also observed at some places of anterior chamber angle by ultrasound biomicroscopy

Anterior segment photography showed mild iridal atrophy of both eyes, which subsequently lead to insufficiently dilated pupils with diameters no more than 5 mm. White cataract was observed in the right eye (Fig. 1). Inspection by anterior segment optical coherence tomography (AS-OCT) *(Cornea/Anterior Segment OCT SS-1000, Tomey Corporation, Japan)* indicated bilateral augmented anterior chambers with backward iridal concavity on horizontal orientation, although the backward concavity in the right eye was markedly reduced before the surgery because of the swelling cataractous lens; while on vertical orientation iris revealed rather flat (Fig. 2a-d). Ultrasound biomicroscopy (UBM) *(MEDA MD-300 L)* showed opacified lens with the apparently elongated suspensory ligaments in the right eye. Partially peripheral anterior synechiae and pectinate ligaments at anterior chamber angle were also observed in both eyes. Ciliary processes were small and scleral processes were not apparent under UBM inspection (Fig. 3a-b).

On the basis of the above findings, the patient was diagnosed with bilateral anterior megalophthalmos complicating white cataract in the right eye.

### Surgical procedure

The operation was performed by an experienced surgeon (Y.L.). A 2.6 mm temporal clear corneal incision was made under topical anesthesia. Viscoelastic agent (DisCoVisc, Alcon, Fort Worth, TX, USA) was then instilled to maintain the anterior chamber with small pupil. Following a continuous curvilinear capsulorhexis of 5.5 mm in size, hydrodissection, chopping, nucleus rotation, and phacoemulsification (CENTURION Vision System, Alcon, Fort Worth, TX, USA) were then performed. A 1-piece foldable IOL (+ 20.5 D, Human Optics PC Acrylic IOL, MC X11 ASP) with four frame haptics to increase intracapsular stability, was inserted into the capsular bag. After aspiration of residual viscoelastic, the incision was hydrated with balanced salt solution and checked for water tightness. Gentle operation was emphasized intraoperatively considering zonular weakness and the deep anterior chamber as the infusion bottle height was set to 75 cm and the phacoemulsification was carried out in a slow-motion mode (vacuum: 300 mmHg; aspiration flow rate: 28 cc/min). Postoperatively, Cravit Eye Drops (*Alcon Laboratories, Inc., Fort Worth, TX, USA*), Pred Forte Eye Drops (*Allergan Pharmaceuticals, Inc., Dublin, Ireland*), and Diclofenac Sodium Eye Drops (*Shenyang Xingqi Pharmaceutical Co. Ltd, Shenyang, China*), all 3 times a day for 4 weeks, were given.

### Postoperative follow-up

**Fig. 4:**
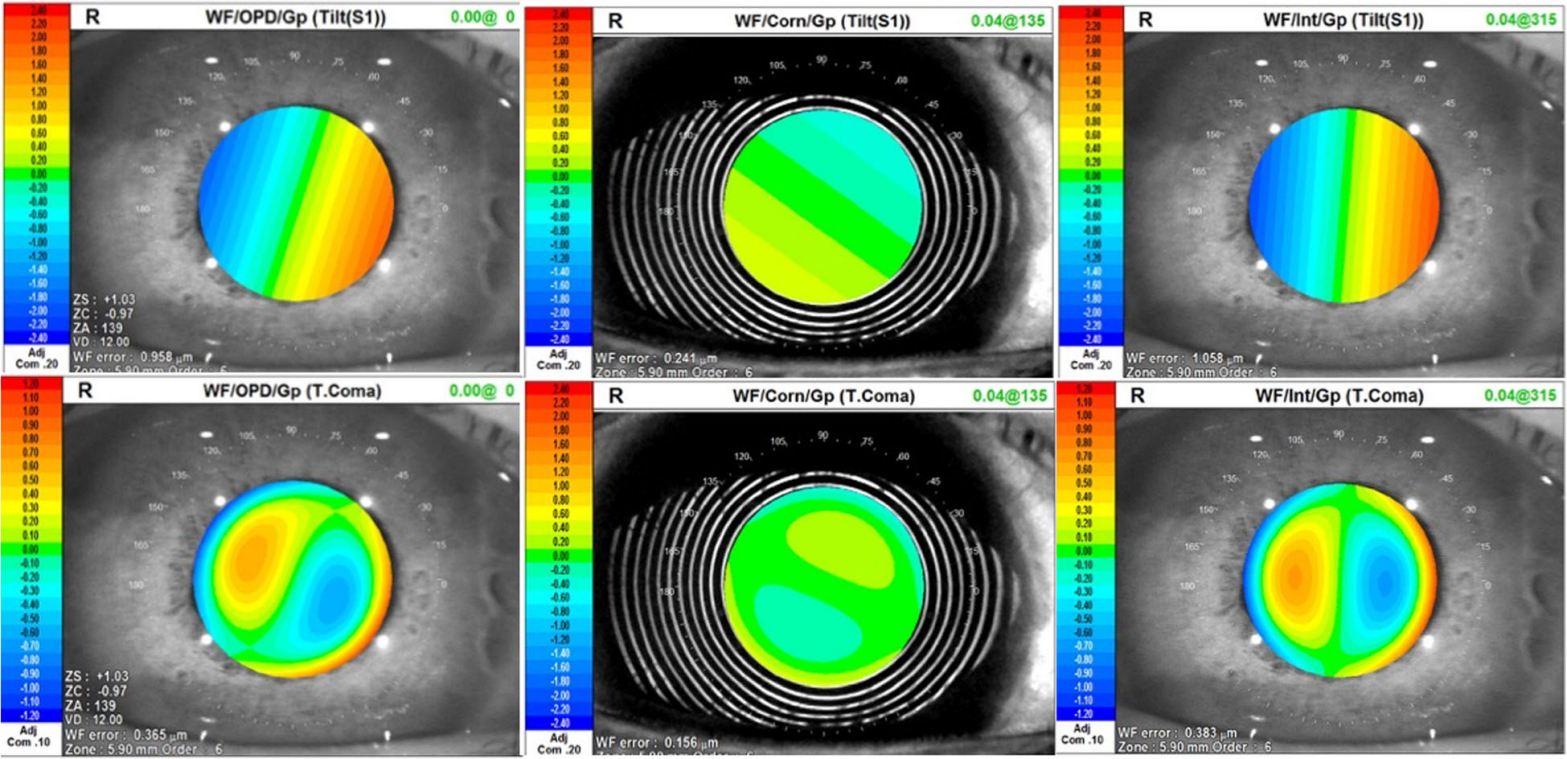
Postoperative OPD-Scan III aberrometry of the right eye. Main sources of increased coma and tilt aberrations were inside of the eye

One month after the surgery, the uncorrected visual acuity (UCVA) and BCVA of the right eye improved to 20/25 and 20/20. The actual postoperative refraction was + 1.50/− 0.50 × 115 in the right eye and the actual postoperative spherical equivalent (SE) was + 1.25 D. The IOP of the right eye was 18 mmHg. Postoperative inspection by AS-OCT indicated a low degree of IOL decentration and iridal backward concavity still remained on horizontal orientation with flat iris on vertical orientation (Fig. 2e, f). OPD-Scan III aberrometry *(Nidek Co, Ltd, Gamagori, Japan)* verified increased internal coma and tilt aberrations indicating slight dislocation of IOL after surgery (Table 2, Fig. 4). Three months after the surgery, the visual acuity and the refractive status of the operated eye were stable and the IOP remained within normal range (15.0 mmHg OD and 17.8 mmHg OS). The IOL also showed good centering and stability during the follow-up.

**Table 2:** Postoperative OPD-Scan III aberrometry results of the right eye

	Zernike/OPD	Zernike/Corn	Zernike/Int
Tilt	0.958@342	0.234@234	1.058@355
High	0.467	0.337	0.514
T. Coma	0.365	0.156	0.383
T. Trefoil	0.256	0.130	0.166
T. Sph	0.041 (C12–0.039)	0.237 (C12 + 0.236)	0.283 (C12–0.282)

The postoperative refractive error equals to the actual postoperative SE (+ 1.25 D) minus the predicted refraction calculated by IOL power calculation formulas as following:

$$ \mathrm{Postoperative}\ \mathrm{Refractive}\ \mathrm{Error}=\mathrm{Actual}\ \mathrm{Postoperative}\ \mathrm{SE}-\mathrm{Predicted}\ \mathrm{Refraction}; $$

In this case, we originally used the SRK/T formula for IOL power calculation and the postoperative refractive error turned out to be + 1.44 D during the follow-up. To find the most accurate power calculation formula with lowest postoperative refractive error for the challenging anterior megalophthalmos cases, we further applied the Haigis and Holladay II formulas (Table 3).

**Table 3:** Comparisons of different IOL calculation formulas

IOL calculation formulas	SRK/T	Holladay II	Haigis
Predicted refraction (D)	−0.19	+ 0.62	+ 1.30
Actual postoperative spherical equivalent (D)^a^	+ 1.25	+ 1.25	+ 1.25
Postoperative refractive error (D) (Postoperative Refractive Error = Actual Postoperative Spherical Equivalent – Predicted Refraction)	+ 1.44	+ 0.63	−0.05
IOL degree when target diopter equals to 0 (D)	20.5	21.5	22.5

^a^Actual postoperative spherical equivalent of our case was + 1.25 D in postoperative follow-up

The postoperative refractive error decreased to a rather low value as − 0.05 D after using the Haigis formula. As to the Holladay II formula, the postoperative refractive error was + 0.63 D. Therefore, compared to the SRK/T and the Holladay II formulas, the Haigis formula may be a more suitable choice according to our case, with higher accuracy and lower postoperative refractive error concerning to the IOL calculation in anterior megalophthalmos.

### Genetic examination

For genetic analysis, we obtained the blood samples of the patient, her son and her parents for whole-exome sequencing based on family to identify any gene mutations directly related to anterior megalophthalmos. However, no known or newfound related gene mutations were found (Table 4).

**Table 4:** Genes and systemic diseases associated with anterior megalophthalmos and megalocornea

Anterior megalophthalmos		Megalocornea	
Gene	Phenotype	Gene	Phenotype
Not Applicable	Trisomy 21	CHRDL1	X-linked Megalocornea
FBN1	Marfan’s Syndrome	SH3PXD2B	Frank-ter Haar Syndrome
FGFR2	Apert syndrome	NOTCH2	Hajdu-Cheney Syndrome
GNPTAB	Mucolipidosis Type 2	PIK3R1	SHORT Syndrome
ISPD, B3GNT1, POMT1, POMT2, ISPD, FKTN, FKRP	Walker Warburg Syndrome	ZNF469	Brittle Cornea Syndrome 1

## Discussion and conclusions

In this challenging case, we successfully performed the phacoemulsification and IOL implantation for a congenital anterior megalophthalmos patient with white cataract. Postoperative examinations showed relatively good visual outcomes with a lower degree of IOL dislocation comparing with existing reports. This is also the first report comparing the different IOL power calculation formulas in anterior megalophthalmos.

All the clinical phenotypes in this case were in accordance with the previously reported diagnostic criteria of anterior megalophthalmos, including bilaterally enlarged corneal diameter (more than 13 mm), deepened anterior chambers, elongated ciliary ring, atrophic iris, overstrained suspensory ligaments, and premature cataract [9]. In addition, corneal thickness, IOP and AL are supposed to be roughly normal. As to genetic factors, 50% of this congenital disease transmits with X-linked recessive inheritance and men consequently constitute most of the patients [10]. We suppose that the present case may follow some other hereditary pattern, which means, it could be resulted from autosomal transmission as the patient was a female. Therefore, we diagnosed the patient as anterior megalophthalmos.

During routine clinical practice, we should also pay attention to other possible diagnosis with enlarged corneal diameters to prevent misdiagnosis. The differential diagnosis of anterior megalophthalmos includes megalocornea, congenital glaucoma and keratoglobus. Megalocornea, mostly followed the X-linked recessive inheritance, has no other anomalies similar to anterior megalophthalmos except for corneal enlargement. Keratoglobus is featured with extensive corneal thinning with globular protrusion and high myopia [2, 10–12]. Congenital glaucoma usually presents with elevated IOP, enlarged cornea with Haab’s striae and abnormal development of anterior chamber angle [13–16]. Therefore, we could have a more affirmative diagnosis as anterior megalophthalmos in this challenging case after excluding other possibilities.

After diagnosis, another preoperative challenge for anterior megalophthalmos was IOL power calculation. The differences of accuracy between the SRK/T formula and Haigis formula may generate from their respective parameter inclusion. As for SRK/T formula and some other commonly used formulas (Holladay I formula, Hoffer Q formula), the calculation of IOL power is mainly based on some given IOL constants, central corneal power and AL [14, 17]. However, as to the Haigis formula, an additional ocular parameter as ACD is included in the calculation [15, 17]. For anterior megalophthalmos, one of its symbolic anatomical deformities is deep anterior chambers and taking this parameter into account renders the Haigis formula to be one of the most reliable power formulas as we also proved this in our case. Besides, the Holladay II formula also includes ACD as a calculating parameter [18], but the postoperative refractive error as + 0.63 D in our case, showing a relatively lower suitability than the Haigis formula. Regardless of ACD, corneal curvature is also an important factor in IOL calculation. The corneas of anterior megalophthalmos were reported to be normal (40.0 D – 45.0 D, 95% CI: 42.84 ± 0.044 D) or slightly flatter [7, 19–23]. Our patient also had relatively flat corneas (Table 1). Previous studies showed that the Haigis formula had higher accuracy than the SRK/T formula for K values less than 43.00 D [24, 25], which was also confirmed in our case. Therefore, the Haigis formula may be more suitable and accurate for the IOL calculation in anterior megalophthalmos with the smallest postoperative refractive errors. Further study on anterior megalophthalmos with a larger sample size will be needed in the future for evidence-based verification.

The next precarious procedure after IOL power calculation is cataract surgery. Enlarged ACD and anterior chamber width could cause the surgical landmarks and dimensions to be abnormal, which further brings difficulty in estimating capsulorhexis size. Difficulty in preoperative pupil dilation resulted from iridal atrophy, as presented in this case, could make the surgery more challenging for the decrease of operating area. Deficient fixation and traction of capsule can also give rise to common postoperative complications as postoperative IOL dislocation, especially when the IOL implanted is not of proper size or the surgical procedure is not even [26, 27].

Negative influences of visual quality brought by IOL dislocation should not be overlooked. Mild or inconspicuous IOL dislocation could presented as monodiplopia and ghosting, indicating impaired visual function. Therefore, confronting this challenge, the cautious selection on the types of IOLs is necessary. We selected MC X11 ASP IOL *(HumanOptics AG, Germany)*, which is lighter than normal with four frame haptics providing strong and firm support within the capsular bag for stable centering. Some recent researches have verified better stability of IOLs with four haptics than those with two haptics. This phenomenon may result from large posterior capsular contact of IOL and constant tension of zonular fibers brought by haptics, which together lead to good IOL centering with less dislocation [28, 29]. Several special IOLs including iris-claw IOL, iris fixation posterior chamber-IOL (PC-IOL) and IOL with stabilizing haptics should be recommended to enhance the stability [30, 31]. Some surgical improvements like suspensory IOL implantation and IOL clamping at capsulorhexis are also helpful for visual rehabilitation [32].

In this case, IOL dislocation with iridal backward concavity still remained on horizontal orientation with flat iris on vertical orientation was observed 1 month after surgery. Unlike previously reports, we firstly applied OPD-Scan III aberrometry in addition to AS-OCT for the evaluation of IOL position, showing a low degree of IOL dislocation as tilt and decentration in the horizontal orientation with increased internal coma aberration in the right eye. Increased high order aberrations disenable light rays to describe a good retinal image for decreased image contrast and limited range of spatial frequencies, disturbing the further detailed visual processing and visual quality finally [33, 34]. Owing to the match of inserted IOL in our case, the IOL dislocation was of a low degree, and postoperative visual rehabilitation revealed relatively good.

As a hereditary disease with unclear mechanism, more than half of the previously reported cases are considered to follow X-linked recessive inheritance, while 40% of the patients inherited autosomally, and only less than 10% of the patients are idiopathic cases [10]. In this case, the gene analysis based on family by whole-exome sequencing showed no known or newfound disease-causing gene mutations directly related to anterior megalophthalmos or megalocornea (Table 4) [35–47]. Atypical size of capsule, idiopathic occurrence and unknown pathogenic location are some possible reasons for the gene sequencing result.

We described the clinical manifestation of a challenging anterior megalophthalmos case and the subsequent surgical managements in addition to the follow-up examinations. Our reflection and management of the challenges during the treatment were illustrated in detail. Finally, our patient showed relatively good visual outcomes with a low degree of IOL dislocation. To our knowledge, this is the first case report of anterior megalophthalmos with a comparison of the different IOL power calculation formulas. We also firstly applied the OPD Scan III aberrometry for postoperative examinations on IOL dislocation in this disease. In conclusion, when encountered with challenging anterior megalophthalmos cases, selections of special IOLs should be highlighted to enhance the stability. Although we concluded that the Haigis formula is probably more accurate for the IOL calculation compared to the SRK/T and the Holladay II formulas in this case, it will have to be replicated with a larger sample size in our future study.

## Data Availability

All data supporting the findings are contained within the manuscript.

## Abbreviations

ACD: Anterior chamber depth
AL: Axial length
AS-OCT: Anterior segment optical coherence tomography
CCT: Central corneal thickness
IOL: Intraocular lens
IOP: Intraocular pressure
LT: Lens thickness
PC-IOL: Posterior chamber intraocular lens
SE: Spherical equivalent
UBM: Ultrasound biomicroscopy
WTW: White to white
